# Maxadilan Prevents Apoptosis in iPS Cells and Shows No Effects on the Pluripotent State or Karyotype

**DOI:** 10.1371/journal.pone.0033953

**Published:** 2012-03-23

**Authors:** Zhiyi Zhao, Rongjie Yu, Jiayin Yang, Xiaofei Liu, Meihua Tan, HongYang Li, Jiansu Chen

**Affiliations:** 1 Ophthalmology Department, First Affiliated Hospital of Jinan University, Guangzhou, China; 2 Institute of ophthalmology, Medical College, Jinan University, Guangzhou, China; 3 Key Laboratory for Regenerative Medicine of Ministry of Education, Jinan University, Guangzhou, China; 4 Bio-engineering Institute of Jinan University, Jinan University, Guangzhou, China; 5 Key Laboratory of Regenerative Biology, South China Institute for Stem Cell Biology and Regenerative Medicine, Guangzhou Institutes of Biomedicine and Health, Chinese Academy of Sciences, Guangzhou, China; University of Newcastle upon Tyne, United Kingdom

## Abstract

Pituitary adenylate cyclase-activating polypeptide (PACAP) is a structurally endogenous peptide with many biological roles. Maxadilan, a 61-amino acid vasodilatory peptide, specifically activates the PACAP type I receptor (PAC1). Although PAC1 has been identified in embryonic stem cells, little is known about its presence or effects in human induced pluripotent stem (iPS) cells. In the present study, we investigated the expression of PAC1 in human iPS cells by reverse transcriptase polymerase chain reaction (RT-PCR) and western blot analysis. To study the physiological effects mediated by PAC1, we evaluated the role of maxadilan in preventing apoptotic cell death induced by ultraviolet C (UVC). After exposure to UVC, the iPS cells showed a marked reduction in cell viability and a parallel increase of apoptotic cells, as demonstrated by WST-8 analysis, annexin V/propidium iodide (PI) analysis and the terminal transferase dUTP nick end labeling (TUNEL) assay. The addition of 30 nM of maxadilan dramatically increased iPS cell viability and reduced the percentage of apoptotic cells. The anti-apoptotic effects of maxadilan were correlated to the downregulation of caspase-3 and caspase-9. Concomitantly, immunofluorescence, western blot analysis, real-time quantitative polymerase chain reaction (RT-qPCR) analysis and *in vitro* differentiation results showed that maxadilan did not affect the pluripotent state of iPS cells. Moreover, karyotype analysis showed that maxadilan did not affect the karyotype of iPS cells. In summary, these results demonstrate that PAC1 is present in iPS cells and that maxadilan effectively protects iPS cells against UVC-induced apoptotic cell death while not affecting the pluripotent state or karyotype.

## Introduction

Traditional stem cell therapies face various impediments, including the ethical and immunological challenges to clinical application. In 2006, Takahashi and Yamanaka published an article in *Cell* that ushered in a new era of stem cell research. Through the retrovirus-mediated transfection of four transcription factors (Oct4, SOX2, c-Myc, and Klf-4), they successfully reprogrammed murine fibroblasts into a state that was similar to an embryonic stem cell [1], a type of reprogrammed cell termed an induced pluripotent stem (iPS) cell. These iPS cells were difficult to distinguish from embryonic stem (ES) cells in morphology, proliferative abilities, surface antigens, gene expression, epigenetic status of pluripotent cell-specific genes and telomerase activity [2]. The generation of iPS cells has provided great promise for studying human diseases without provoking ethical and immunological problems. In addition to *in vitro* disease modeling, these cells could be utilized for many toxicological and pharmaceutical applications. The potential use of iPS cells, which can be generated from any patient to produce genetically identical pluripotent cells or patient-specific cells for therapy, has provoked enormous investigative interest within the scientific community. Although substantial progress has been made over the past few years to characterize iPS cells and the techniques used to culture iPS cells have greatly improved, iPS cells remain vulnerable to undergoing apoptosis [3]. The identification of an anti-apoptotic drug that can effectively prevent apoptosis in the iPS cell culture medium will be important for generating iPS cells at a scale that can accommodate future clinical applications.

Pituitary adenylate cyclase-activating polypeptide (PACAP) is a bioactive peptide isolated from ovine hypothalamic tissues with two bioactive forms, consisting of either 38 (PACAP-38) or 27 (PACAP-27) amino acid residues. PACAP exerts its actions through at least three distinct receptors: PACAP receptor 1 (PAC1), VIP receptor 1 and VIP receptor 2 [4]. Maxadilan, a 61-amino acid vasodilatory peptide, was initially isolated from the salivary glands of the sand fly *Lutzomyia longipalpis*. Although it shares no significant sequence homology with PACAP, maxadilan has been shown to be a PAC1-specific agonist, thereby serving as a useful tool to investigate the functions of PACAP mediated through PAC1 in diverse physiological settings [5]. PACAP and its receptor PAC1 can protect cells from apoptosis. Kanekar S et al. [6] reported that both PACAP and maxadilan could prevent TNFα-mediated cell death in olfactory placodal cells and that PACAP protects the mouse olfactory epithelium cells against axotomy-induced apoptosis. Racz B et al. [7] reported that PACAP effectively protects cochlear cells against oxidative stress-induced apoptotic cell death. Gasz B et al. [8] showed that PACAP was able to attenuate oxidative stress-induced cardiomyocyte apoptosis. In 2004,Cazillis M et al. [9] demonstrated that PAC1 is expressed and functional in mouse embryonic stem (ES) cells. Soon after, Hirose M et al. [10] also identified that PAC1 is present in ES cells. However, little is known about the presence and effects of PAC1 in iPS cells. In this study, the expression or absence of PAC1 in iPS cells was investigated, and maxadilan was subsequently used to probe the anti-apoptotic effects mediated by PAC1 in iPS cells. This research attempted to understand if maxadilan could be an additive to facilitate large-scale culturing of iPS cells.

## Materials and Methods

### Cell culture conditions and drug treatments

The UMC human iPS cell line was used in all experiments. This iPS cell line was established from the umbilical cord matrix and amniotic membrane mesenchymal cells by transduction of retroviral factors, including Oct4, SOX2, c-Myc, and Klf4 [11]. The cells were cultured under feeder-free culture conditions. Briefly, iPS cells were cultured in mTeSR1 medium (STEM CELL) on dishes coated with Matrigel (Sigma-Aldrich). The cells were grown in 5% CO_2_ with 95% humidity. The cell medium was changed daily, and spontaneously differentiated colonies were removed when appropriate. iPS cells were passaged every six days with 0.05% trypsin-EDTA (STEM CELL) at 37°C for 3–5 min. When colonies near the edge of the plate began to dissociate from the bottom, the enzyme was removed, and colonies were washed with mTeSR1 medium. Cells were collected by gently pipetting and replated onto fresh Matrigel-coated dishes. The ROCK inhibitor Y-27632 (10 µM) was added to each well for the first day after each passage.

### Western blot analysis

PAC1 was detected by western blot analysis in iPS cells. iPS cells were pretreated with 100 nM of maxadilan for 24 h and passaged 3 times without removing the spontaneously differentiated colonies prior to quantitative western blot analysis for Nanog, OCT4 and SOX2 protein levels. This same procedure was used on control iPS cells that were not treated with maxadilan. iPS cells (at a density of 1×10^6^ cells) were lysed with RIPA buffer containing a protease inhibitor cocktail (Bocai Biotechnology) and sonicated on ice. The sonicated material was then centrifuged for 20 min at 15,000×rpm at 4°C, and the supernatant was collected. Fifty Micrograms of total protein as determined by the BCA method (Pierce) were separated on 10% SDS-PAGE gels and transferred onto a PVDF membrane (Bio-Rad). The membrane was incubated overnight at 4°C with the following primary antibodies: rabbit polyclonal anti-PAC1 antibody (1∶2000, Santa Cruz), rabbit polyclonal anti-OCT4 antibody (1∶1000, Cell Signaling), rabbit polyclonal anti-Nanog antibody (1∶1000, Cell Signaling), rabbit monoclonal anti-SOX2 antibody (1∶1000, Cell Signaling) and rabbit monoclonal anti-β-actin antibody (1∶3000, Cell Signaling). The membrane was incubated with the goat anti-rabbit IgG secondary antibody (1∶3000; Santa Cruz) for 1 h at room temperature. After adding the ECL chemiluminescence reagent (Pierce), the membrane was incubated with developer solution for 1 min and with a fixative for 0.5 min. Quantitation of band intensities was performed by scanning the immunostaining band (Tanon2500)and analyzing the image with ImageJ 1.39 software.

### Viability of iPS cells after UVC irradiation

One hundred microliters of the single-cell iPS cell suspension (1×10^4^ cells) were seeded onto each well of a 96-well plate coated with Matrigel. iPS cells were cultured in mTeSR1 medium in 96-well plates to produce colonies at 80%–90% confluence. Ten microliters of maxadilan (at 0 nM, 3 nM, 5 nM, 10 nM, 20 nM, 30 nM and 50 nM) [12] were added to each well, and the plates were incubated at 37°C for 1 h. Cells were washed with phosphate buffered solution (PBS) and subsequently exposed to 50 J/m^2^, 75 J/m^2^ and 100 J/m^2^ ultraviolet C (UVC) at 254 nm (SPECTRONICS, EBF-260C). Fresh culture medium and the appropriate concentrations of maxadilan were added, and cells incubated for 24 h. Control wells contained iPS cells cultured in mTeSR1 medium and were not irradiated with UVC. iPS cell viability was measured by WST-8 analysis using the Cell Counting Kit-8 (CCK-8) (Dojindo). The samples were stained with 10 µl of the CCK-8 solution and incubated on the plate in a CO_2_ incubator for 3 h. Absorbance (OD) of the iPS cells at a wavelength of 450 nm was spectrophotometrically measured with a microplate reader equipped with the Magellan (5.0) software (Tecan Safire2, Switzerland).

### Annexin V and propidium iodide (PI) assays

iPS cells were cultured in mTeSR1 medium in 6-well plates to produce colonies at 80%–90% confluence. The iPS cells were irradiated with UVC as described above. The UVC+30 nM maxadilan iPS samples were treated with 30 nM of maxadilan for 1 h prior to exposure to 100 J/m^2^ UVC, and the UVC+0 nM maxadilan iPS samples were exposed to 100 J/m^2^ UVC in the absence of maxadilan. After iPS cells were exposed to UVC, fresh culture medium and the appropriate concentration of maxadilan were added to each well, and the cells were incubated for 7 h. Control wells containing iPS cells were cultured in mTeSR1 medium without UVC irradiation. To detect early apoptotic activity, an Annexin V-FITC/PI Apoptosis Detection Kit (KeyGEN) was used according to the manufacturer's instructions. iPS cells were washed with cold PBS and added to 200 µl of the Annexin V-binding buffer. After the samples were stained with 2 µl of FITC-labeled Annexin V and 2 µl of PI, the samples were immediately analyzed by flow cytometry (BD FACSAria™).

### Terminal transferase dUTP nick end labeling (TUNEL) assays

iPS cells were cultured in mTeSR1 medium in 6-well plates to produce colonies at 80%–90% confluence and irradiated with UVC as described above. The UVC+30 nM maxadilan iPS sample was treated with 30 nM maxadilan for 1 h prior to exposure to 100 J/m^2^ UVC, whereas the UVC+0 nM maxadilan iPS group was exposed to 100 J/m^2^ UVC without pretreatment with maxadilan. After the iPS cells were exposed to UVC, fresh culture medium and the appropriate concentration of maxadilan were added to each well, and the cells were incubated for 9 h. Control wells contained iPS cells cultured in mTeSR1 medium and did not receive UVC radiation. To assess cellular apoptosis, a one-step TUNEL Assay Kit was used according to the manufacturer's instructions (KeyGEN). Biotinylated fluorescein-dUTP was incorporated into late-stage fragmented DNA using terminal deoxynucleotidyl transferase, and the fluorescein was measured using a fluorescence plate reader (Multilabel Counter 1420, VICTOR3V, Perkin Elmer). An excitation wavelength of 480 nm and an emission wavelength of 520 nm were utilized.

### Assessment of caspase-3 and caspase-9

To identify the signaling mechanism by which maxadilan protects against UVC-induced cell death, we measured the activity of caspase-3 and caspase-9 in iPS cells treated with maxadilan after UVC irradiation. iPS cells were cultured in mTeSR1 medium in 6-well plates to produce colonies at 80%–90% confluence. The UVC+30 nM maxadilan iPS cells were treated with 30 nM of maxadilan for 1 h prior to exposure to 100 J/m^2^ UVC, whereas the UVC+0 nM maxadilan iPS cells were exposed to 100 J/m^2^ UVC without any pretreatment with maxadilan. After the iPS cells were exposed to UVC, fresh culture medium and the appropriate concentration of maxadilan were added to each well, and the cells were incubated for 6 h. Control wells containing iPS cells were cultured in mTeSR1 medium and did not undergo UVC irradiation. iPS cells were measured by a caspase-3 and caspase-9 Colorimetric Assay Kit (KeyGEN) and the BCA Protein Assay Kit (KeyGEN) according to the manufacturer's instructions. For analysis of caspase activity, cells were lysed for 60 min on ice in lysis buffer, and 50 µl of the reaction buffer was added to 50 µl of the cellular supernatant solution (containing 50 µg of soluble protein) and further incubated with 5 µl of caspase-3 and caspase-9 substrates for 4 h. Absorbance (OD) was read spectrophotometrically using a microplate reader (Tecan Safire2, Switzerland). Excitation and emission wavelengths were set at 400 and 500 nm, respectively.

### Karyotype analysis

iPS cells were incubated with 100 nM maxadilan (the optimal concentration of maxadilan to promote proliferation of iPS cells, data not shown) for 24 h on day 5 after passaging. The iPS cells were subsequently passaged three times without removing the spontaneously differentiated colonies. iPS cells that were not treated with maxadilan served as the control. iPS cells were incubated with 0.05 mg/ml of colcemid (Invitrogen) for 150 min at 37°C in a 5% CO_2_ incubator. Cells were washed with PBS and trypsinized for 2 min at room temperature. Cells were fixed in methanol/glacial acetic acid (3∶1) three times and then dropped onto slides for chromosome spreads. The slides were baked overnight at 55°C, treated with 0.05% trypsin for 30 s and stained with Giemsa solution.

### Reverse Transcription Polymerase Chain Reaction (RT-PCR) and Real-Time Quantitative Polymerase Chain Reaction (RT-qPCR) analysis

PAC1 was detected by RT-PCR in iPS cells. iPS cells were pretreated with 100 nM of maxadilan for 24 h and passaged 3 times without removing the spontaneously differentiated colonies prior to RT-qPCR analysis for *OCT4*, *Nanog*, *SOX2*, *Rex1*, *UTF1*, *TERT*, *NESTIN* and *PAX6* gene expression levels. This same procedure was used on control iPS cells that were not pretreated with maxadilan. Primer sequences are shown in Table 1. Total RNA from iPS cells was isolated using TRIzol, and the resulting RNA samples were quantified by measuring the OD at 260 nm; the OD 260/280 ratios for all RNA samples were between 1.8 and 2.1. Total RNA (2 µg) was reverse transcribed in a 20 µl reaction mixture containing 4 µl of 5× Reverse Transcriptase Buffer, 2 µl dNTPs, 1 µl RNase inhibitor, 1 µl oligo-dT, 1 µl AMV Reverse Transcriptase, 9 µl DEPC H_2_O, and 200 U of Reverse Transcriptase (M-MLV) at 42°C for 1 h. The cDNA was synthesized, diluted and used for RT-PCR for PAC1 andβ-actin. Total cDNA was used to perform qPCR on the CFX96 Real-Time PCR Detection System (Bio-Rad). The reaction mixture consisted of 12.5 µl SYBR® *Premix Ex Taq*™ (2×), 0.5 µl forward and reverse primers (10 µM), 2 µl diluted cDNA and 9.5 µl ddH_2_O. The reaction conditions were 95°C for 30 s, followed by 40 cycles of 95°C for 5 s, 60°C for 30 s. The relative expression of the genes was normalized against GAPDH or β-actin. Melting curves were examined for the quality of the PCR amplification of each sample, and quantification was performed using the comparative CT (2^−ΔΔCT^) method [13].

**Table 1:** List of primers.

Primers	Sequences (5′to 3′)	GeneBank Number
GAPDH total-F	AGAAGGCTGGGGCTCATTTG	BC059110
GAPDH total-R	AGGGGCCATCCACAGTCTTC	
β-actin total-F	TGAAGTGTGACGTGGACATC	NM_001101.3
β-actin total-R	GGAGGAGCAATGATCTTGAT	
PAC1 total-F	GGGTTCCTATTGGTTAGTTGGT	NM_001118.4
PAC1 total-R	GGCTGGTGTTTAGACAGAGTTCC	
Nanog total-F	CAAGAACTCTCCAACATCCTGAAC	NC_000001
Nanog total-R	CTGCGTCACACCATTGCTATTC	
OCT4 total-F	GAAGGATGTGGTCCGAGTGT	NC_000006
OCT4 total-R	GTGAAGTGAGGGCTCCCATA	
SOX2 total-F	ATGCACCGCTACGACGTGA	NM_003106.3
SOX2 total-R	CTTTTGCACCCCTCCCATTT	
REX1 total-F	AAACGGGCAAAGACAAGACAC	NM_174900.3
REX1 total-R	ATAGCACACATAGCCATCACATAA	
UTF-1 total-F	CGCCGCTACAAGTTCCTTAAA	NM_003577.2
UTF-1 total-R	GGATCTGCTCGTCGAAGGG	
TERT total-F	CGGAAGAGTGTCTGGAGCAA	NM_001193376.1
TERT total-R	GGATGAAGCGGAGTCTGGA	
NESTIN total-F	AACAGCGACGGAGGTCTCTA	NC_000012
NESTIN total-R	TTCTCTTGTCCCGCAGACTT	
PAX6 total-F	TTGCTGGAGGATGATGACAGAGGAA	NM_001604
PAX6 total-R	TTCTGCATGCTGGCTCTGGCT	
SOX1 total-F	AACCAGGACCGGGTCAAACGG	NM_005986
SOX1 total-R	CGCTTCTCGGCCTCGGACATG	
PPAR total-F	CACACACCGAGGACTCTTGCG	NC_000022
PPAR total-R	AACAAACACGCGCGTCTCCGT	
GATA4 total-F	ACTCGGAGCTTCTCCGCCTTTG	NC_000008
GATA4 total-R	GGCCCGTCAGTCCCGGTAAC	
FOXA2 total-F	GAGGGAGGCGACAGCGTTAGCA	NC_000020
FOXA2 total-R	TGTCCCGCAGGTTGCTTGCTG	
SOX17 total-F	CGCACGGAATTTGAACAG	NC_000008
SOX17 total-R	CAGTAATATACCGCGGAGCTG	

### *In vitro* differentiation

To examine *in vitro* differentiation, iPS cells treated with 100 nM maxadilan for 24 h were cultured using a 24-well plate with ultra-low adhesiveness to produce embryoid bodies (EBs) in suspension. The EBs were subsequently cultured in differentiation medium, which consisted of 80% DMEM/F12, 20% Knockout Serum Replacement, 1 mM L-glutamine, 0.1 mM β-mercaptoethanol and 0.1 mM non-essential amino acids (Gibco). Control iPS cells were not treated with maxadilan. iPS cells aggregated and generated EBs for 18 days. The attached EBs expanded and differentiated into cells with various morphological features after being seeded onto Matrigel-coated plates and cultured for 20 days. RT-PCR was performed for the markers of ectoderm (*SOX1*, *NESTIN* and *PAX6*), mesoderm (*GATA4* and *PPAR*) and endoderm (*FOXA2* and *SOX17*), and RT-qPCR was performed for *NESTIN* and *PAX6* gene expression levels in these cells as described above.

### Immunofluorescence assay

iPS cells were treated with 100 nM maxadilan for 24 h and passaged 3 times without removing the spontaneously differentiated colonies before the immunofluorescence assay was performed. Control iPS cells were not treated with maxadilan. The qualitative detection of Nanog, OCT-4, SOX2, SSEA-4 and TRA-1-60 was determined by immunofluorescence utilizing a fluorescence microscope (Leica DMRA, Germany), whereas the quantitative detection of Nanog, OCT-4 was determined using a microplate reader (Tecan Safire2, Switzerland). Excitation and emission wavelengths were set at 495 and 520 nm, respectively, and the fluorescence absorption (OD) was measured. Briefly, after fixation in 4% paraformaldehyde for 30 min at room temperature, iPS cells were permeabilized with 0.1% Triton-X 100 in Dulbecco's Phosphate Buffered Saline (DPBS) for 15 min at room temperature, washed three times with DPBS and incubated with DPBS containing 10% fetal bovine serum for 30 min at room temperature. The cells were incubated with the primary antibodies [rabbit polyclonal anti-OCT4 antibody (1∶400, Cell Signaling), rabbit polyclonal anti-Nanog antibody (1∶800, Cell Signaling), rabbit monoclonal anti-SOX2 antibody (1∶400, Cell Signaling), mouse monoclonal anti-SSEA-4 antibody (1∶1000, Cell Signaling), mouse monoclonal anti-TRA-1-60(S) antibody (1∶1000, Cell Signaling), rabbit polyclonal anti-AQP1 antibody (1∶400; Santa Cruz) served as a negative control] for 60 min, and then with the secondary antibodies [mouse anti-rabbit IgG secondary antibody (1∶400; Santa Cruz), Goat anti-mouse IgG secondary antibody (1∶100; Bioss), Goat anti-mouse IgM secondary antibody (1∶100; Bioss)] for 60 min before staining with DAPI.

### Statistical Analysis

Statistical analysis was performed with a software package (SPSS16.0). The statistical significance comparing multiple sample sets with the control was analyzed with a one-way ANOVA followed by the Dunnett's test. Comparisons between the two groups were analyzed using Student's *t*-tests. A *p*-value of less than 0.05 was considered statistically significant. Data are presented as the means ± SEM. All results were derived from three independent experiments.

## Results

### Analysis of PAC1 in iPS cells

**Figure 1:**
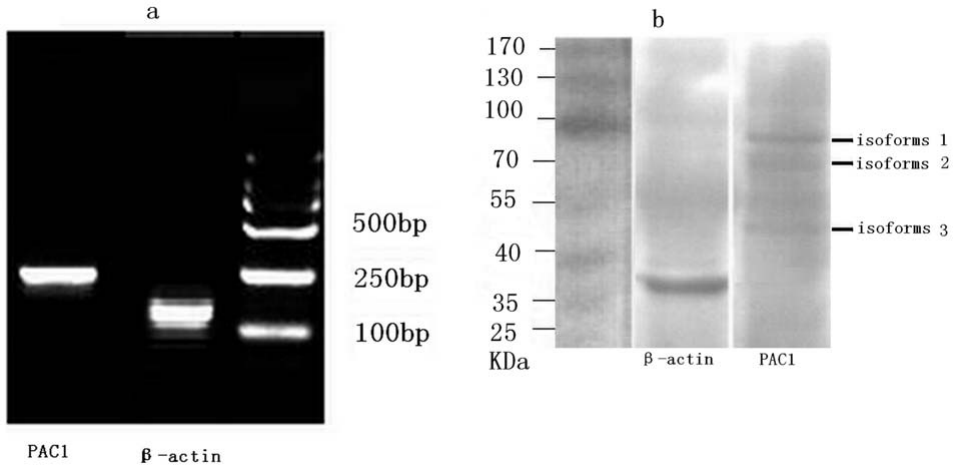
PAC1 mRNA and protein were expressed in iPS cells. The expression of PAC1 mRNA in iPS cells by RT-PCR analysis (a). The expression of PAC1 protein in iPS cells by western blot analysis showed that there are three kinds of PAC1 isoforms (b). The molecular weights of these PAC1 isoforms (1, 2 and 3) vary from about 50 kDa to 80 kDa.

To determine if PAC1 was present in iPS cells, RT-PCR and western blot analyses were performed. The primer sequences of *PAC1* were shown in Table 1. As shown in figure (Figure 1), *PAC1* mRNA was clearly expressed in iPS cells and the results of western blot indicated that iPS cells had three kinds of PAC1 isoforms. The molecular weights of these PAC1 isoforms (1, 2 and 3) vary from about 50 kDa to 80 kDa.

### Test of iPS cell viability after UVC irradiation

**Figure 2:**
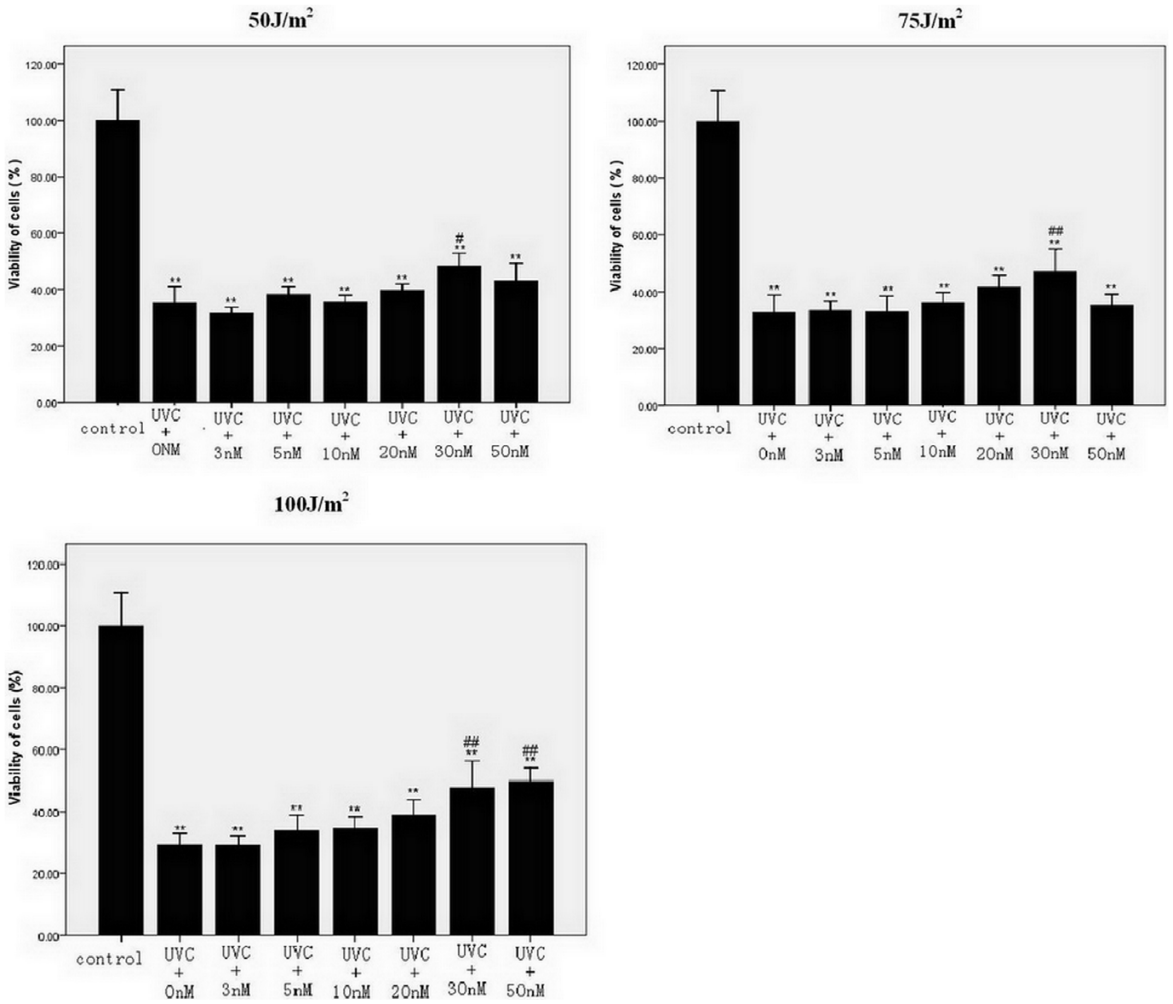
Effects of maxadilan at various concentrations in iPS cells exposed to UVC. Cell viability in iPS cells that were exposed to 50 J/m^2^, 75 J/m^2^ and 100 J/m^2^ UVC and treated with maxadilan (at 0 nM, 3 nM, 5 nM, 10 nM, 20 nM, 30 nM and 50 nM) was detected by WST-8 analysis. iPS cells without UVC irradiation were used as control. Values are expressed as the mean of OD ± SEM of three independent experiments. ** *P*<0.01 *vs.* control group using Dunnett's test. #*P*<0.05 or ##*P*<0.01 *vs.* UVC+0 nM Maxadilan group using Dunnett's test.

Maxadilan markedly affected cell survival after UVC irradiation (Figure 2). iPS cells that were exposed to 50 J/m^2^, 75 J/m^2^ and 100 J/m^2^ UVC and treated with 30 nM maxadilan showed a significant increase in cell viability compared with iPS cells that were not treated with maxadilan. iPS cells that were irradiated with 50 J/m^2^, 75 J/m^2^ and 100 J/m^2^ UVC showed a 64.63%, 67.23% and 70.8% reduction in cell viability, respectively, compared with the control group. The addition of 30 nM maxadilan resulted in a 51.75%, 53.1% and 52.43% decrease in cell viability, respectively, compared with the control group. iPS cells exposed to 100 J/m^2^ UVC and treated with 50 nM of maxadilan also showed a significant increase in cell viability compared with iPS cells that were not treated with maxadilan. The viability of iPS cells after irradiation with 100 J/m^2^ UVC was reduced by 70.8% compared with the control group, and the addition of 50 nM maxadilan displayed a 50.29% decrease compared with the control group.

### Annexin V and PI assays

**Figure 3:**
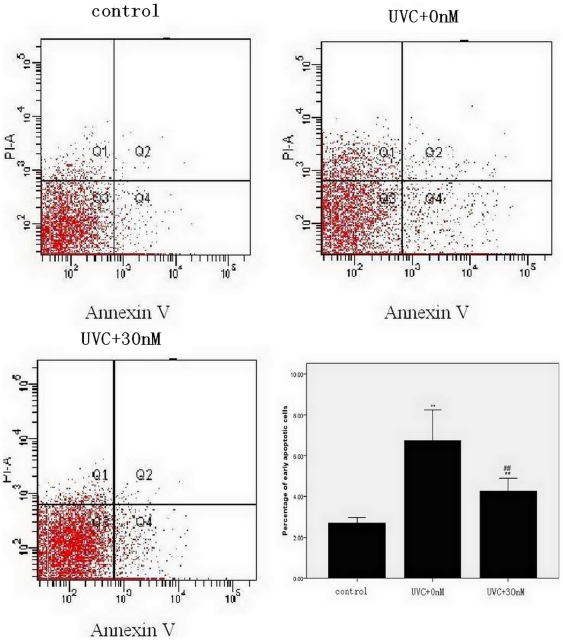
Flow cytometric analysis of iPS cell apoptosis with various treatments. Percentage of early apoptotic cells in iPS cells that were exposed to 100 J/m^2^ UVC and treated with maxadilan (at 0 nM and 30 nM) were measured by flow cytometric analysis. iPS cells that were not irradiated with UVC were used as control. The Q2 area represents cell necrosis, the Q3 area represents living cells and the Q4 area represents early apoptotic cells. Data are expressed as the mean of percentage ± S.E.M of three independent experiments. **P<0.01 *vs.* control group using Dunnett's test. # #*P*<0.01 *vs.* UVC+0 nM maxadilan group with the Student's *t*-test.

Annexin V and PI were analyzed by flow cytometry to detect apoptosis in iPS cells cultured under various treatments. During the early stages of apoptosis, cells typically have an intact cell membrane that are not stained with PI; however, externalization of phosphatidylserine (membrane phospholipids) can be detected by annexin V. Using this method, we found that the addition of 30 nM maxadilan to iPS cells irradiated with 100 J/m^2^ UVC dramatically reduced the ratio of early apoptotic cells compared with iPS cells without maxadilan treatment. iPS cells irradiated with 100 J/m^2^ UVC showed a 248% increase in the ratio of early apoptotic cells compared with the control group, whereas cells treated with 30 nM maxadilan under the same conditions displayed a 158% increase compared with the control group (Figure 3 ).

### TUNEL assays

**Figure 4:**
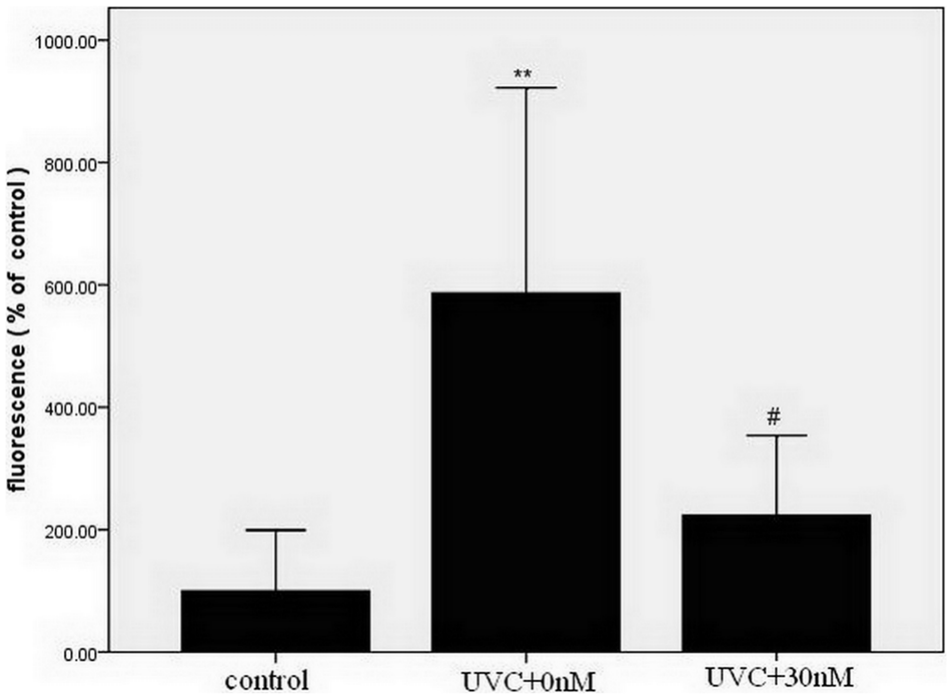
Comparison of Fluorescein-dUTP in iPS cells with various treatments by TUNEL assay. Fluorescein-dUTP of iPS cells that were exposed to 100 J/m^2^ UVC and treated with maxadilan (at 0 nM and 30 nM) was detected by TUNEL assays. iPS cells that did not receive UVC radiation were used as control. Fluorescence values are given in arbitrary units and are expressed as the mean ± S.E.M of three independent experiments. **P<0.01 *vs.* control group using Dunnett's test. #*P*<0.05 Vs UVC+0 nM maxadilan group with the Student's *t*-test.

A TUNEL assay was performed to assess the anti-apoptotic effects of maxadilan in iPS cells irradiated with UVC. The values of biotinylated fluorescein-dUTP were proportional to the volume of fragmented DNA in apoptotic cells. Our data revealed that the addition of 30 nM maxadilan to iPS cells irradiated with 100 J/m^2^ UVC dramatically reduced the percentage of apoptotic cells compared with iPS cells that were not treated with maxadilan. iPS cells irradiated with UVC showed a 587% increase in the percentage of apoptotic cells compared with control group, and the addition of 30 nM maxadilan displayed only a 224% increase in apoptotic cells compared with the control group (Figure 4).

### Caspase-3 and caspase-9 assays

**Figure 5:**
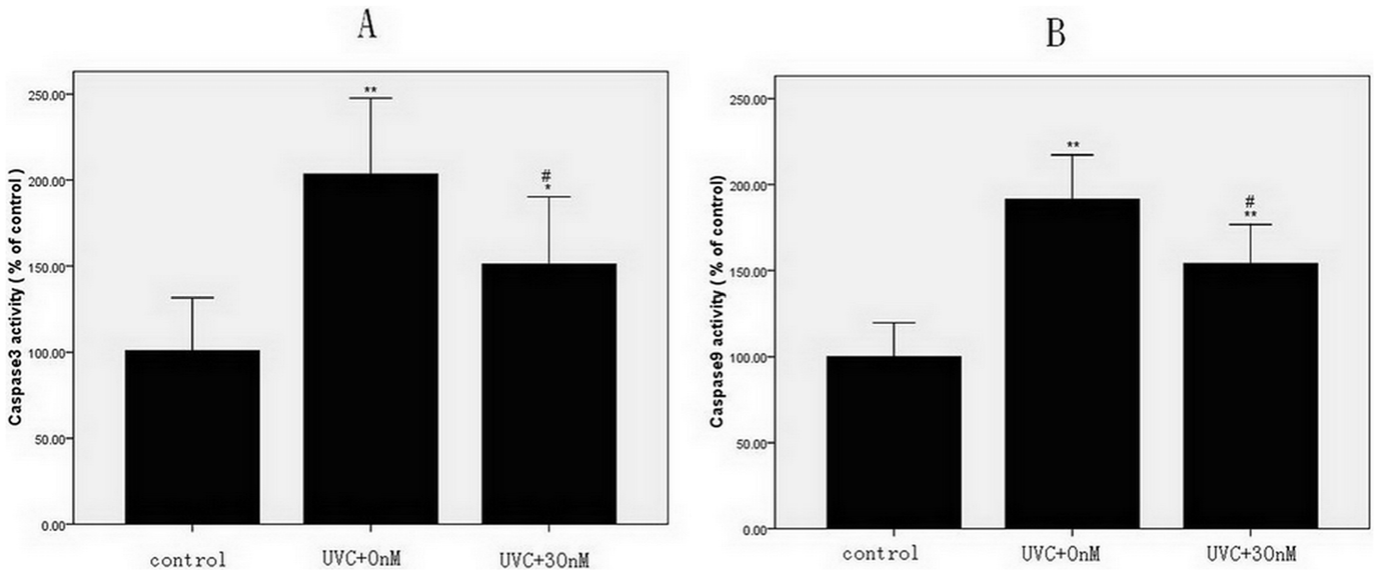
Assessment of caspase-3 and caspase-9 activities in iPS cells with various treatments. Caspase-3 activity (A) and caspase-9 activity (B) in iPS cells that were exposed to 100 J/m^2^ UVC and treated with maxadilan (at 0 nM and 30 nM) were measured by caspase-3 and caspase-9 treatment. iPS cells that did not undergo UVC irradiation were used as control. The results are expressed as the mean of OD ± S.E.M of three independent experiments.*P<0.05 or **P<0.01 *vs.* control group using Dunnett's test. #*P*<0.05 *vs.* UVC+0 nM maxadilan group using the Student's *t*-test.

To analyze the apoptotic machinery of iPS cells induced by UVC and the anti-apoptotic machinery of maxadilan, caspase-3 and caspase-9 assays were performed. Our data showed that the addition of 30 nM maxadilan to iPS cells irradiated with 100 J/m^2^ UVC significantly downregulated caspase-3 and caspase-9. iPS cells irradiated with 100 J/m^2^ UVC showed a 104% and 92% increase in activity of the caspase-3 and caspase-9, respectively, compared with the control group, and the addition of 30 nM maxadilan displayed a 51% and 54% increase, respectively, compared with the control group (Figure 5).

### Karyotype analysis of iPS cells

Karyotype analysis was performed to determine the effect of maxadilan on the karyotype of iPS cells. Karyotype analysis of iPS cells treated with 100 nM maxadilan revealed a normal chromosome complement of 46XX.

### RT-PCR and RT-qPCR analysis

**Figure 6:**
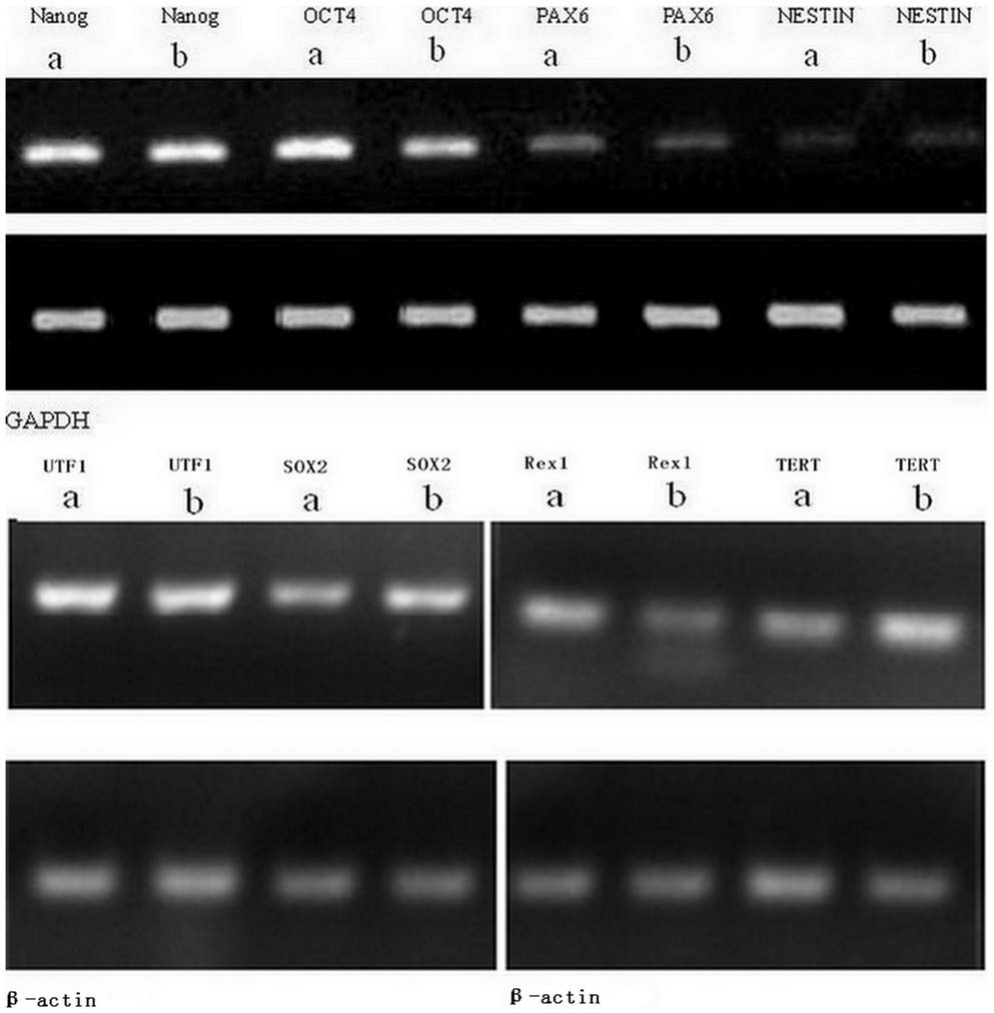
RT-PCR analysis of mRNA expression in iPS cells with or without maxadilan. RT-PCR analysis of Nanog, OCT4, PAX6, NESTIN, UTF1, SOX2, Rex1, and TERT mRNA expression in iPS cells of both the control group (a) and iPS cells treated with 100 nM maxadilan (b). iPS cells that were not treated with maxadilan served as the control.

**Figure 7:**
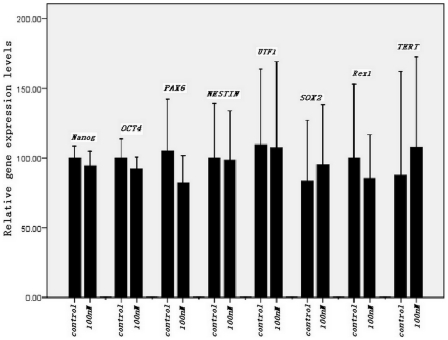
RT-qPCR analysis of mRNA expression in iPS cells with or without maxadilan. Comparison of relative gene expression levels of Nanog, OCT4, PAX6, NESTIN, UTF1, SOX2, Rex1 and TERT in iPS cells between the control group and iPS cells treated with 100 nM maxadilan by RT-qPCR analysis. iPS cells that were not treated with maxadilan served as the control. Values are expressed as the mean of relative gene expression levels ± S.E.M of three independent experiments. There are no significant differences between the two groups (*p*>0.05 with the Student's *t*-test).

To understand the effect of maxadilan on the pluripotent state of iPS cells and to determine if maxadilan produces neuronal differentiation of iPS cells, we used RT-qPCR analysis to quantitatively compare the gene expression levels of *Nanog*, *OCT4*, *SOX2*, *Rex1*, *UTF1*, *TERT*, *NESTIN* and *PAX6* between control iPS cells and cells treated with 100 nM maxadilan. Our data showed no significant difference in the gene expression levels of *Nanog*, *OCT4*, *SOX2*, *Rex1*, *UTF1*, *TERT*, *NESTIN* and *PAX6* between the two groups (Figure 6 and 7).

### Western blot analysis

**Figure 8:**
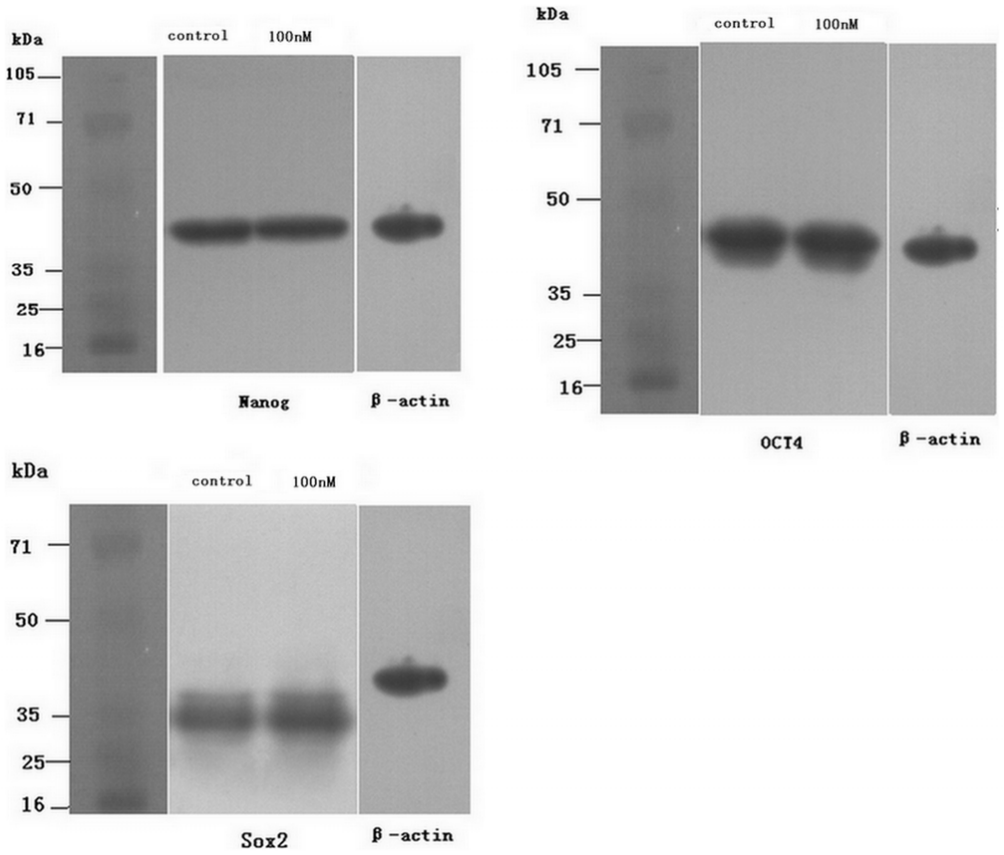
Western blot analysis in iPS cells with or without maxadilan. Western blot analysis of Nanog, OCT4 and SOX2 protein expression in iPS cells of both the control group and iPS cells treated with 100 nM maxadilan. iPS cells that were not treated with maxadilan served as the control. The results are representative of three independent experiments.

**Figure 9:**
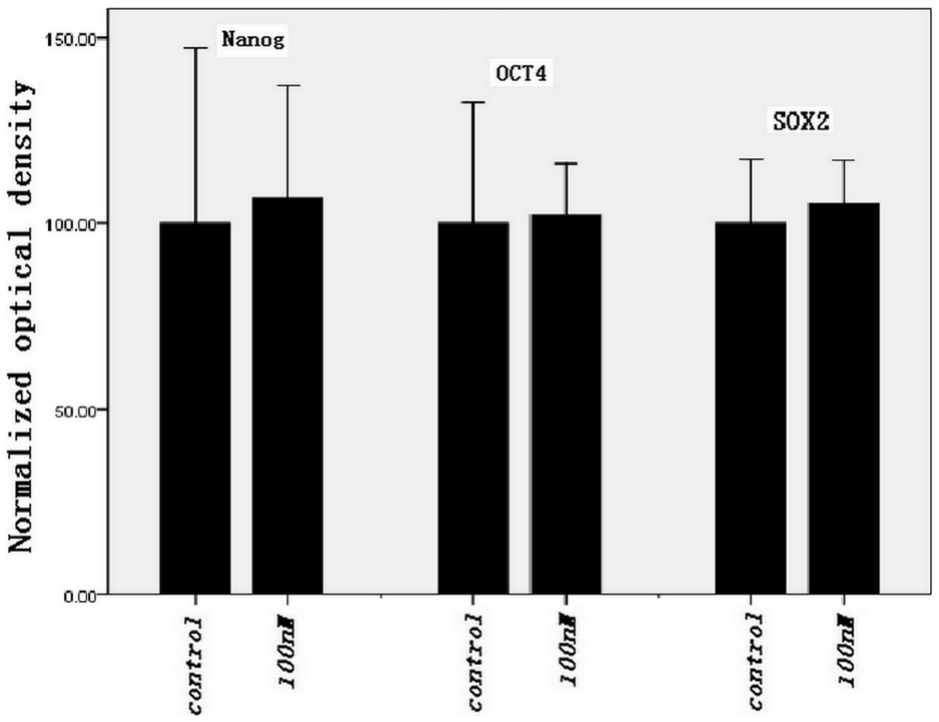
Comparison of protein expression levels in iPS cells with or without maxadilan. Comparison of protein expression levels of Nanog, OCT4 and SOX2 in iPS cells between the control group and iPS cells treated with 100 nM maxadilan by western blot analysis. iPS cells that were not treated with maxadilan served as the control. Results are normalized to β-actin. Values are expressed as the mean ± S.E.M of three independent experiments. There are no significant differences between the two groups (p>0.05 with the Student's t-test).

To determine the effect of maxadilan on the pluripotent state of iPS cells, we used western blot analysis to quantitatively compare the protein levels of Nanog, OCT4 and SOX2 between control iPS cells and cells treated with 100 nM maxadilan. Our data showed that Nanog, OCT4 and SOX2 protein were clearly expressed in iPS cells (Figure 8) and that there was not significant difference in the protein levels of Nanog, OCT4 and SOX2 between the two groups (Figure 9).

### *In vitro* differentiation

**Figure 10:**
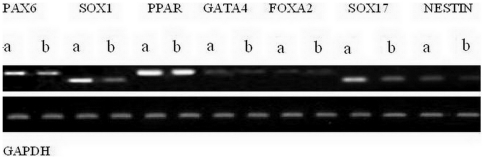
RT-PCR analysis of mRNA expression in EBs from iPS cells with or without maxadilan. RT-PCR analysis the mRNA levels of PAX6, SOX1, PPAR, GATA4, FOXA2, SOX17 and NESTIN in the EBs from the control group (a) and from iPS cells treated with 100 nM maxadilan (b). iPS cells that were not pretreated with maxadilan served as the control.

**Figure 11:**
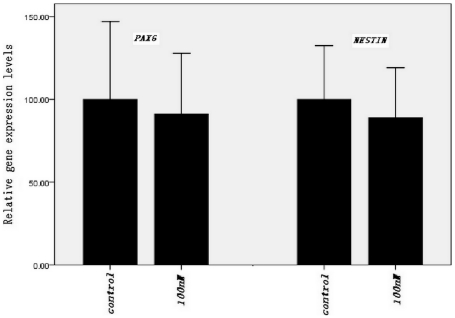
RT-qPCR analysis of mRNA expression in EBs from iPS cells with or without maxadilan. RT-qPCR analysis comparing the relative gene expression levels of PAX6 and NESTIN in the EBs from the control group and from iPS cells treated with 100 nM maxadilan. iPS cells that were not pretreated with maxadilan served as the control. Values are expressed as the mean of relative gene expression levels ± S.E.M of three independent experiments. There are no significant differences between the two groups (*p*>0.05 with the Student's *t*-test).

To characterize the ability of iPS cells treated with maxadilan to differentiate *in vitro*, RT-PCR was used to measure the mRNA levels of *PAX6*, *SOX1*, *PPAR*, *GATA4*, *FOXA2*, *SOX17* and *NESTIN* in cells of EBs from both the control group and the group treated with 100 nM maxadilan. Our data showed that both of iPS cells treated with maxidalan and their nontreated counterparts had the ability to form EBs and further differentiate. The differentiated cells from both groups expressed *SOX1*, *PAX6*, *GATA4*, *PPAR*, *FOXA2*, *SOX17 and NESTIN*, which are important markers of three embryonic layers (Figure 10). To determine whether maxadilan could produce neuronal differentiation of iPS cells, we analyzed the gene expression levels of *NESTIN* and *PAX6* by RT-qPCR in control EBs or those treated with 100 nM maxadilan. Our data showed that there was no significant difference in the gene expression levels of *NESTIN* or *PAX6* in the EBs between the control group and the maxadilan-treated group (Figure 11).

### Immunofluorescence assay

**Figure 12:**
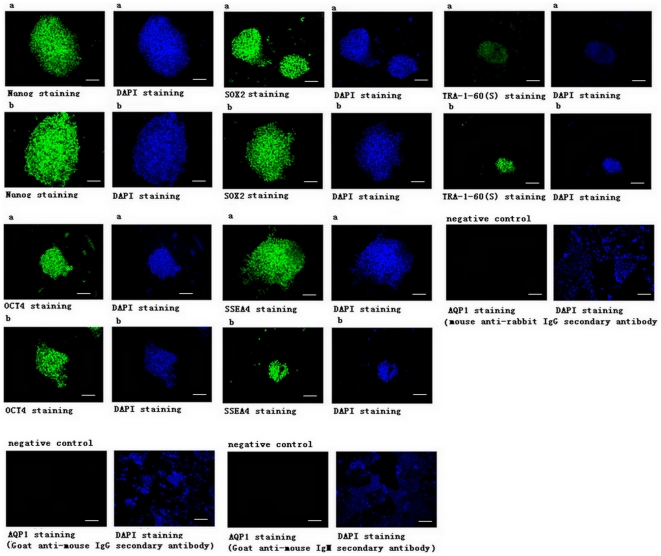
Immunofluorescence staining in iPS cells with or without maxadilan. Immunofluorescence examining the protein expression of Nanog, OCT4, SOX2, SSEA-4 and TRA-1-60(s) in iPS cells of both the control group (a) and in iPS cells that had been treated with 100 nM maxadilan (b). iPS cells that were not treated with maxadilan served as the control. iPS cells treated with rabbit polyclonal anti-AQP1 antibody served as a negative control. Mouse anti-rabbit IgG secondary antibody, goat anti-mouse IgG secondary antibody and goat anti-mouse IgM secondary antibody were respectively used as secondary antibodies in this experiment. All scale bars are 100 µm.

**Figure 13:**
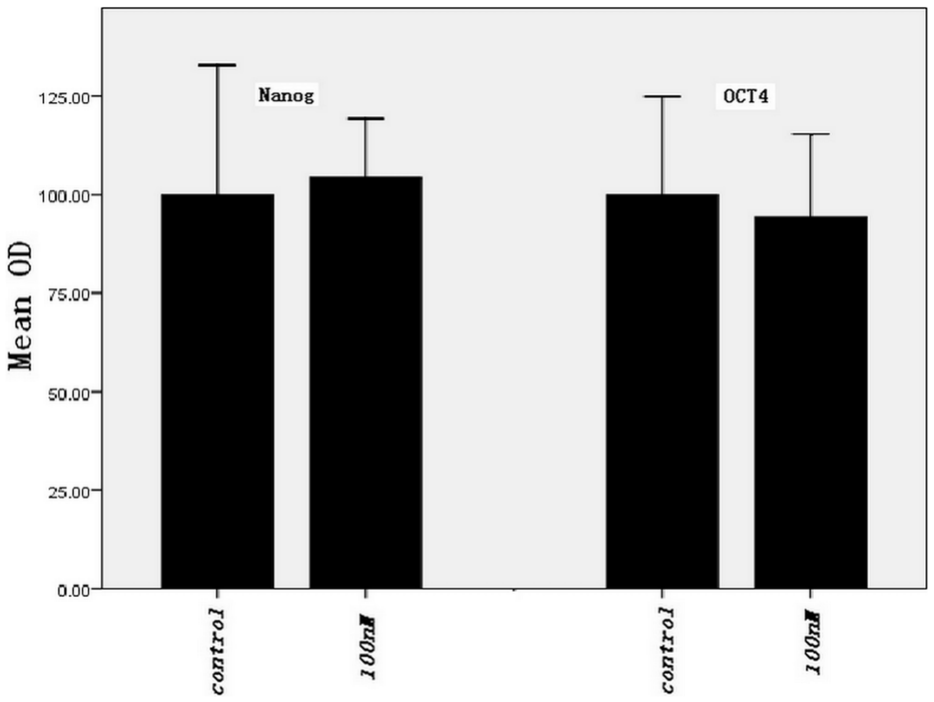
Comparison of protein fluorescence of Nanog and OCT4 in iPS cells with or without maxadilan. Comparison of fluorescence OD values of Nanog and OCT4 in iPS cells of the control group and in iPS cells treated with 100 nM maxadilan by microplate reader analysis. iPS cells that were not treated with maxadilan served as the control. Values are expressed as the mean of OD ± SEM of three independent experiments. There are no significant differences between the two groups (*p*>0.05 with the Student's *t*-test).

To determine the effect of maxadilan on the pluripotent state of iPS cells, we examined the protein expression levels of Nanog, OCT-4, SOX2, SSEA-4 and TRA-1-60 by immunofluorescence in control cells and in cells treated with 100 nM maxadilan. We found that both the control group and the group treated with 100 nM maxadilan expressed Nanog, OCT-4, SOX2, SSEA-4 and TRA-1-60(S) and maintained the characteristics of undifferentiated stem cells (Figure 12). A quantitative assay of Nanog and OCT4 protein levels also showed no significant differences in expression between the two groups (Figure 13).

## Discussion

In recent years, there has been significant advancement in the technical aspects used to culture iPS cells. However,there is a problem not yet resolved, which iPS cell culture conditions are still limited by the low survival rate that commonly follows enzymatic dissociation and iPS cells are vulnerable to several kinds of apoptosis, including detachment-induced apoptosis (anoikis) [14] and dissociation-induced apoptosis (nonanoikis) [15]. This characteristic is an obstacle for the development of techniques to manipulate and batch produce iPS cells. Some studies have shown promising results to circumvent the problem of apoptosis in iPS cell culture. Pakzad M et al. [14] reported that the addition of the ROCK inhibitor Y-27632 to the extracellular matrix has both anti-apoptotic effects and augments effect on cells. This group concluded that the addition of Y-27632 to the extracellular matrix could increase the plating efficiency of iPS cells during passage. Ohgushi M et al. [15] showed that nonanoikis, caused by ROCK-dependent hyperactivation of actomyosin in iPS cell culture, could be efficiently inhibited by the myosin inhibitor blebbistatin. Wang X et al. [16] found that iPS cells were subjected to continuous anoikis in culture, and anoikis could be inhibited by the addition of bFGF to the medium. The identification of an anti-apoptotic drug that is effective when added to iPS cell culture medium will help to increase the scale of iPS cell culture, which will be critical for future clinical applications.

PACAP is a member of the vasoactive intestinal peptide (VIP)/secretin/glucagon family of peptides. PACAP and VIP belong to the same family of peptides and have well-established trophic properties through shared G protein-coupled receptor signaling [4]. PACAP has at least three distinct receptors: PACAP receptor 1 (PAC1), VIP receptor 1 and VIP receptor 2. In 2004,Cazillis M et al. [9] demonstrated that PAC1 was functionally expressed in mouse ES cells and that PACAP may induce the differentiation of ES cells into a neuronal phenotype. Subsequently, Hirose M et al. [10] also showed that PAC1 was present in undifferentiated ES cells. They found that the expression of PAC1 mRNA was further upregulated after terminal differentiation into neurons, and the expression of PAC1 mRNA markedly decreased after glial differentiation. Utilizing an electrophysiological patch-clamp technique, Chafai M et al. [17] reported in 2006 that PACAP and VIP, which act via the PAC1 and VPAC2 receptors, facilitated the generation of electrical activity in differentiating ES cells. However, little is known about the presence and effects of PAC1 in iPS cells. In this study, RT-PCR analysis demonstrated clearly that PAC1 was present in human iPS cells. PAC1 belongs to the B class of GPCR (G Protein Coupled Receptor). The PAC1 gene contains more than 18 exons, and alternative splicing of two regions (the first extracellular (EC1) domain and/or the third intracellular cytoplasmic (IC3) loop) of the PAC1 gene results in a relatively large number of PAC1 isoforms, whose molecular weights varied from about 50 kDa to 80 kDa [18]. In this study, the western-blot result showed that iPS cells had three kinds of PAC1 isoforms. It has been known that the structural divergency of GPCRs, as a result of alternative splicing, can influence a number of receptor properties, including ligand affinity, G-protein coupling, and the regulation of intracellular signaling [19]. So we need more assays to show the role of each isoform in mediating effects the maxadilan on iPS cells.

PACAP and its receptor PAC1 are involved in cell proliferation, differentiation and viability. For example, many studies have reported that PACAP can protect cells from apoptosis, which involves various kinds of cell lines, such as the olfactory epithelium and olfactory placodal cells [6], cochlear cells [7], cardiomyocytes [8], and cortical neurons [20]. After confirming the presence of PAC1 in iPS cells, one of our priorities was to determine whether PACAP could protect iPS cells from apoptosis. Therefore, we utilized maxadilan, a PAC1-specific agonist, to investigate the anti-apoptotic functions of PACAP. In this study, we found that maxadilan provided significant protection of iPS cells from apoptosis induced by UVC irradiation.

Apoptosis is a basic feature of all animal cells and is essential for normal development and tissue homeostasis. However, despite the fact that most apoptotic programs lead to similar morphological and biochemical endpoints, the apoptotic machinery is variable [21]. Identifying the apoptotic machinery of iPS cells induced by UVC irradiation and the anti-apoptotic machinery that is affected by maxadilan will help to define iPS applications. Among the various molecules that take part in the apoptotic process, caspase plays an important role during the initiation and effector phase of apoptotic cell death. Caspase is secreted as an inactive protease and can be activated autocatalytically, thereby unleashing the “caspase cascade” that amplifies the apoptotic signal. Caspase-3 is a prototypical caspase and an important protease for the execution of apoptosis [22]. Martin SA et al. [23] found that when an apoptosis model was induced by UV, caspase-3 was activated by the UV-induced apoptosis pathway. Woo M et al. [24] reported that caspase-3-deficient ES cells were resistant to apoptosis induced by UV irradiation. These results demonstrate that caspase-3 is likely necessary for apoptosis following UV irradiation. Moreover, in response to most apoptotic stimuli, multiple caspases are interrelated and affect one another. For instance, caspase-9 is one component of a complex that is critical for caspase-3 activation. By activating caspase-3, caspase-9 becomes the upstream member of the apoptotic protease cascade [25]. Hakem R et al. [26] found that both caspase-9^−/−^ and caspase-3^−/−^ ES cells were resistant to apoptotic signals induced by UV irradiation. They concluded that UV irradiation preferentially triggered the activation of an apoptotic pathway involving caspase-9 and caspase-3 in ES cells. In our study, iPS cells irradiated with UVC showed a significant increase in caspase-3 and caspase-9 activities compared with the control group. We conclude that caspase-3 and caspase-9 are involved in iPS cell apoptosis induced by UVC. Alleviation or inhibition of caspase activity by the use of physiological or pharmacological agents has been known to reduce apoptosis [27]. Studies have demonstrated that PACAP can prevent apoptosis and inhibit caspase-3 activation in another apoptotic model and cell type [8], [28]. We found that UVC irradiation led to a marked increase in caspase-3 and caspase-9 activations and that maxadilan counteracted this effect. Our observations strongly suggest that maxadilan protects iPS cells from apoptosis. The anti-apoptotic role of maxadilan acts, at least in part, by reducing the activation of caspase-3 and caspase-9. However, the detailed anti-apoptotic signaling mechanisms of maxadilan are not fully elucidated.

PACAP is a neurotrophic peptide. Several studies have concluded that the PACAP/PAC1 system promoted neuronal or astrocyte differentiation of neural progenitor cells (NPCs) [29]. However, others have shown that PACAP peptides are downregulated, preventing differentiation of NPCs and maintaining their multipotent state [30]. Cazillis et al. [9] demonstrated that PACAP could induce the differentiation of ES cells into a neuronal phenotype. Therefore, we were interested to determine if maxadilan could produce neuronal differentiation of iPS cells. Both NESTIN and PAX6 are markers of neural progenitor cells, the neuroectoderm and neural crest stem cells. These factors may be utilized as markers of neural differentiation [31]. In this study, *PAX6* gene expression was lower in the iPS cells treated with maxadilan than in the control group, but this difference was not significant. In addition, the gene expression of *NESTIN* in both control iPS cells and the maxadilan treatment group was nearly identical. Moreover, there was not a significant difference between the control and maxadilan-treated iPS cells in the gene expression of *NESTIN* or *PAX6* in cells derived from EBs. These data demonstrated that maxadilan could not produce neuronal differentiation of iPS cells (at least at this particular dosage).

Although mTeSR1 medium, which contains recombinant human basic fibroblast growth factor and recombinant human transforming growth factor β, was used, a very small number of spontaneously differentiated cells were observed during the culture and passage of iPS cells. We observed that the number of spontaneously differentiated cells increased gradually if differentiated colonies were not removed prior to 3 passages of iPS cell culture. To understand the effect of maxadilan on the pluripotent state of iPS cells, we quantitatively compared the relative gene expression levels of *Nanog*, *OCT4*, *SOX2*, *Rex1*, *UTF1* and *TERT* by RT-qPCR in both control iPS cells and the maxadilan treatment group. We also qualitatively examined the protein expression of Nanog, OCT4, SOX2, SSEA-4 and TRA-1-60 while quantitatively compared the protein expression levels of Nanog and OCT4 by immunofluorescence between these two groups. In addition, we used western blot analysis to examine the protein expression of Nanog, OCT4 and SOX2. In this study, there were no significant differences in gene expression of *Nanog*, *OCT4*, *SOX2*, *Rex1*, *UTF1* and *TERT* between the control group and the maxadilan-treated iPS cells. There were also no significant differences in protein expression levels of Nanog, OCT4 by immunofluorescence assay and Nanog, OCT4 and SOX2 by western blot analysis between these two groups. Pluripotency markers, Nanog, OCT4, SOX2, SSEA-4 and TRA-1-60(S) in maxadilan-treated iPS cells were confirmed by immunofluorescence. These results demonstrate that iPS cells may retain characteristics of undifferentiated stem cells even after maxadilan treatment. Moreover, our data showed that both of iPS cells treated with maxidalan and their nontreated counterparts had the ability to form EBs and further differentiate. The differentiated cells from both groups expressed the important markers of three embryonic layers. Our data implied that treatment of iPS cells with maxadilan does not affect their pluripotent state and displayed a normal karyotype.

In conclusion, our results demonstrate that PAC1 is present in human iPS cells. We also showed that maxadilan dramatically increased iPS cell viability and reduced the percentage of apoptotic cells after UVC irradiation. The anti-apoptotic effect of maxadilan was correlated to the downregulation of caspase-3 and caspase-9. Concomitantly, maxadilan did not affect the pluripotent state or karyotype of iPS cells. Our research suggests that maxadilan may be used as an anti-apoptotic additive in iPS cell culture.
